# 

**Published:** 1878-01

**Authors:** 


### CONTENTS:

ARTICLE I.—A Plea for Life Assurance. By Prof. Jas. B. Hodgkin, D. D. S...... 385

ARTICLE II.—Annual Meeting of the Virginia State Dental Association.......... 392

ARTICLE III—New Treatment of Plaster Casts........................ 397

ARTICLE IV.—Nervousness in the Male. By S Weir Mitchell, M D.............. 398

ARTICLE V.—Essay on Cleft Palates. By Dr. Davenport...................... 411

ARTICLE VI.—Hand Pressure and Soft Foil, vs. Malleting and Cohesive Foil. By J. Foster Flagg, D. D. S.............................. 419

ARTICLE VII.—Alumni Association of Baltimore College of Dental Surgery....... 421

### EDITORIAL, ETC.

Dental Education as Seen by a Canada Editor.................................. 422

### BIBLIOGRAPHICAL.

Harris' Medical and Dental Dictionary. Fourth Edition........................ 425

Biddle's Materia Medica. Eighth Edition...................................... 426

Elements of Dental Materia Medica and Therapeutics........................... 426

### MONTHLY SUMMARY.

A New Cause for Alarm, Arsenic in Vulcanite.................................. 427

Specific for Diptheria....................................................... 427

Phosphate of Lime in Relation to Fractures................................... 427

Functions of the Liver....................................................... 428

Preparation of Celluloid..................................................... 428

Colored Borax Varnishes...................................................... 428

Mastic for Fastening India Rubber on Metals.................................. 429

To take Rust out of Steel.................................................... 429

The Dangers of Ether......................................................... 429

Bichromate of Potash as an Antiseptic........................................ 430

Properties of the Human Gastric Juice........................................ 430

Salicylic Acid and Borax..................................................... 430

Nitrate of Amyl.............................................................. 431

Atheroma..................................................................... 431

A Daring Therapeutist........................................................ 432

Scientific American.......................................................... 432

American Agriculturist....................................................... 432

